# Impaired Activation of Visual Attention Network for Motion Salience Is Accompanied by Reduced Functional Connectivity between Frontal Eye Fields and Visual Cortex in Strabismic Amblyopia

**DOI:** 10.3389/fnhum.2017.00195

**Published:** 2017-04-21

**Authors:** Hao Wang, Sheila G. Crewther, Minglong Liang, Robin Laycock, Tao Yu, Bonnie Alexander, David P. Crewther, Jian Wang, Zhengqin Yin

**Affiliations:** ^1^Key Laboratory of Visual Damage and Regeneration and Restoration of Chongqing, Southwest Eye Hospital/Southwest Hospital, Third Military Medical UniversityChongqing, China; ^2^Faculty of Science, School of Psychological Science, Technology and Engineering, La Trobe UniversityMelbourne, VIC, Australia; ^3^Department of Radiology, Southwest Hospital, Third Military Medical UniversityChongqing, China; ^4^Murdoch Children's Research InstituteMelbourne, VIC, Australia; ^5^Centre for Human Psychophysiology, Swinburne University of TechnologyMelbourne, VIC, Australia

**Keywords:** adult strabismic amblyopia, visual attention, attention network, motion salience, FEF

## Abstract

Strabismic amblyopia is now acknowledged to be more than a simple loss of acuity and to involve alterations in visually driven attention, though whether this applies to both stimulus-driven and goal-directed attention has not been explored. Hence we investigated monocular threshold performance during a motion salience-driven attention task involving detection of a coherent dot motion target in one of four quadrants in adult controls and those with strabismic amblyopia. Psychophysical motion thresholds were impaired for the strabismic amblyopic eye, requiring longer inspection time and consequently slower target speed for detection compared to the fellow eye or control eyes. We compared fMRI activation and functional connectivity between four ROIs of the occipital-parieto-frontal visual attention network [primary visual cortex (V1), motion sensitive area V5, intraparietal sulcus (IPS) and frontal eye fields (FEF)], during a suprathreshold version of the motion-driven attention task, and also a simple goal-directed task, requiring voluntary saccades to targets randomly appearing along a horizontal line. Activation was compared when viewed monocularly by controls and the amblyopic and its fellow eye in strabismics. BOLD activation was weaker in IPS, FEF and V5 for both tasks when viewing through the amblyopic eye compared to viewing through the fellow eye or control participants' non-dominant eye. No difference in V1 activation was seen between the amblyopic and fellow eye, nor between the two eyes of control participants during the motion salience task, though V1 activation was significantly less through the amblyopic eye than through the fellow eye and control group non-dominant eye viewing during the voluntary saccade task. Functional correlations of ROIs within the attention network were impaired through the amblyopic eye during the motion salience task, whereas this was not the case during the voluntary saccade task. Specifically, FEF showed reduced functional connectivity with visual cortical nodes during the motion salience task through the amblyopic eye, despite suprathreshold detection performance. This suggests that the reduced ability of the amblyopic eye to activate the frontal components of the attention networks may help explain the aberrant control of visual attention and eye movements in amblyopes.

## Introduction

Amblyopia is a developmental disorder usually clinically diagnosed on the basis of reduced visual acuity through one or both eyes but without any observable retinal pathology (Noorden, 1996). It is associated with early abnormal visual experience (through strabismus, anisometropia, or visual deprivation) during the critical period for vision and is characterized by impaired binocular function, deficient stereopsis and slower and less accurate acquisition of monocular fixation (Asper et al., 2000a; Mckee et al., 2016). Impaired binocular function in strabismus has traditionally been associated with suppression of at least part of the central visual field (Hess, 1991), although recent findings suggest neural plasticity of such suppression and the possibility of treatment (Hess et al., 2014; Kehrein et al., 2016).

Amblyopia affects ~1.6–3.6% of the population (Eibschitz-Tsimhoni et al., 2000; Williams et al., 2001) and until quite recently has been considered unlikely to respond positively to therapy after late childhood. However, over the last decade new techniques including use of video games have been developed that show that many amblyopes have some binocular capacity and that even adult visual acuity can improve with training (To et al., 2011; Hess et al., 2012; Hess and Thompson, 2013, 2015).

Neural mechanisms of strabismic amblyopia have been well studied electrophysiologically in animal models using cat (Hubel and Wiesel, 1965; Cleland et al., 1982; Freeman et al., 1983; Chino et al., 1988; Asper et al., 2000a,b; Crewther and Crewther, 2015) and monkey (Kiorpes et al., 1998; Watanabe et al., 2005; Wong et al., 2005; Bi et al., 2011; Husk et al., 2012). The evidence from the cat studies clearly show that processing of spatial information is normal at the level of the retina and the lateral geniculate nucleus (Cleland et al., 1982; Gillard-Crewther and Crewther, 1988; Crewther and Crewther, 1990). These results confirm earlier reports (Crewther and Crewther, 1993) showing that the few binocular cortical neurons that remain in V1 in strabismic amblyopic cats show lower spatial resolution through the misaligned eye compared to the undeviating fellow eye, whereas the majority of V1 neurons are monocular, indicative of binocular suppression.

As with other phenomena (such as binocular rivalry), single cell studies appear to be at odds with the results of fMRI studies. Miki et al. (2003) and more recently Hess et al. (2009, 2010), have shown deficient activation responses from LGN as well as from early cortical areas, potentially indicative of anomalies of effective connectivity in individuals with amblyopia. The reconciliation of such single cell, local field and BOLD studies still requires resolution in terms of signal transfer in the forward direction and cortical feedback.

Not surprisingly, misalignment of the visual axes has long been known in animal and human studies to be associated with abnormal binocular fixation and eye movements, and deficits in visual attention (Singer, 1982a; Asper et al., 2000b; Thiel and Sireteanu, 2009; Wang et al., 2015; Hou et al., 2016). In fact, Singer (1982b) discussed the role of attention in the development of amblyopia and observed that synchronous binocular eye movements are crucial for the development of normal visual pathways (Ciuffreda et al., 1979a,b). Abnormal eye movements from birth have since been shown to contribute to abnormal development of visual attention (Vedamurthy et al., 2008; Wang et al., 2015) and in turn, visual acuity deficits, though the mechanisms and implications are not yet clear.

Neuroimaging studies have also looked at dorsal stream function during motion processing in amblyopia. Passive viewing of expanding/contracting rings has been associated with less activation in MT+ in participants with amblyopia relative to controls (Bonhomme et al., 2006). Direction discrimination of high-level random-dot kinematograms also produced less activation, relative to a control group, in V3A, MT+, and PPC in both eyes of children with anisometropic and strabismic amblyopia (Ho and Giaschi, 2009). Behavioral impairments in multiple object tracking through both eyes of strabismic and anisometropic amblyopes has also been reported by Secen et al. (2011), with associated imaging data demonstrating reduced BOLD signal change in frontal eye fields (FEF), anterior intraparietal sulcus (IPS) and motion area MT+ but not in area V1, during high attentional load conditions. The subtle impairments in FEF and IPS were argued to reflect a deficiency in higher-level motion systems impacting visual attention networks (Farivar et al., 2011; Secen et al., 2011).

Eye movements are associated with shifts of overt visual attention (Vernet et al., 2014) and can be either involuntary shifts to incoming unexpected moving stimuli (stimulus-driven) or voluntarily driven (goal-directed) sequential and organized shifts in sustained attention, especially during near work such as reading (Amso and Scerif, 2015). The neuroanatomical basis of such eye movements and goal directed attention mechanisms has been well described in humans using brain imaging (PET and fMRI) (Petersen et al., 1994; Corbetta, 1998; Corbetta and Shulman, 2002; Bressler et al., 2008) and in primate multichannel spike and local field potential recordings (Gregoriou et al., 2012). The two major visual attentional networks comprise a bilateral dorsal fronto-parietal goal-directed attention network and a more ventral, right lateralized fronto-parietal network related to salience and reorienting (Corbetta and Shulman, 2002; Howe et al., 2009; Thiebaut De Schotten et al., 2011) that connects with early visual areas V1, V5, superior colliculus, basal ganglia and saccade planning areas in IPS and FEF (Amso and Scerif, 2015). By comparison, fMRI informed EEG in strabismic amblyopia, shows that compared to controls, the modulatory effects of selective attention on the input from the amblyopic eye are substantially reduced in V1 and extrastriate areas hV4 and hMT (Hou et al., 2016).

Thus, to dissociate the involuntary and voluntary attention networks in individuals with strabismic amblyopia, we designed two kinds of visuo-motor tasks to examine the neural loci and efficiency of involuntary motion salience-driven attention and voluntary goal-directed saccadic eye movements in adult strabismic amblyopes and controls. Firstly, a motion-salience attention task utilized the appearance of coherent motion-defined objects within random noise to determine the time required for the perception of a salient object to activate attention, so assessing stimulus-driven visual spatial attention (Alexander et al., 2015). Secondly, a task biased toward voluntary goal-directed saccades that required subjects to quickly plan and saccade to an object appearing randomly in different horizontal locations was employed (Sestieri et al., 2007).

Psychophysical thresholds for motion sensitivity were first measured, and then a suprathreshold version of the task and the goal-directed saccade task were used in the magnet for functional magnetic resonance imaging (fMRI) to test activation and the functional connectivity of attention-and eye movement-related brain areas including primary visual cortex (V1), the motion processing area (V5), oculomotor planning area intraparietal sulcus (IPS), and frontal eye fields (FEF) of the strabismic and fellow eyes of amblyopes and normal controls. To date there have been a few reviews of studies (Li et al., 2011; Parks and Madden, 2013; Hamm et al., 2014) suggesting impaired functional connectivity between visual cortex and extrastriate and parietal areas during motion processing through the amblyopic eye compared to the fellow eye (Thompson et al., 2012), but no comparison of activation during goal-directed as opposed to stimulus-driven attention tasks.

## Materials and methods

### Subjects

Eight adult strabismic amblyopes aged 17–30 years (*M* = 22.63, *SD* = 5.10) and eight adult volunteers aged 24–30 years (*M* = 26.25, *SD* = 1.83) with normal visual function were recruited for this study.

All subjects received detailed eye examinations that included visual acuity, refraction, slit lamp examination, ophthalmoscopy, ocular motility, cover test of both eyes and synoptophore examination of binocular function to ensure suitability for this study. The clinical details of the amblyopic subjects are shown in Table 1. All of the subjects had undergone strabismic surgery for esotropia in infancy, but for subjects 2, 3, 6, and 8 surgery was not totally successful, resulting in exotropic alignment in adulthood. Clinical histories revealed that the amblyopic esotropic eyes of subjects 1, 2, 3, 4, 7, 8 were also anisometropic in childhood. No subject had any history of neurological or psychiatric disorders. Subjects were screened for metal implants to ensure safety associated with fMRI participation.

**Table 1:** **Clinical details of the strabismic amblyopic subjects**.

**Subjects**	**Gender**	**Age**	**Refraction**		**Visual acuity**		Strabismus^*^	
			**Left**	**Right**	**Left**	**Right**	**Present**	**History**
ST1	Male	24	+0.50DS/−0.75DCx75	−1.75DS/−0.50DCx165	20/400	20/20	LES	LES
ST2	Female	17	+1.25DS/−0.75DCx180	−1.25DS/−0.50DCx160	20/400	20/20	LEX	LES
ST3	Male	17	+0.50DS/−0.75DCx35	0.00DS/−1.00DCx5	20/800	20/20	LEX	LES
ST4	Female	30	−1.25DS/−0.50DCx170	+0.50DS/−0.50DCx180	20/20	20/50	RES	RES
ST5	Male	23	−0.50DS/−0.50DCx100	−0.25DS/−0.25DCx100	20/20	20/400	RES	RES
ST6	Female	27	−1.50DS/−0.25DCx140	−1.00DS/−0DC	20/20	20/125	REX	RES
ST7	Male	26	+0.50DS/−0.5 DCx15	+1.25DS/−0.75DCx180	20/20	20/200	RES	RES
ST8	Female	17	+1.50DS/−1.00DCx180	−1.25DS/−0.25 DCx15	20/200	20/20	LEX	LES

* *LES, L eye esotropia; LEX, L eye exotropia; RES, R eye esotropia; REX, R eye exotropia*.

### Psychophysical data acquisition

Eye dominance of each subject was first ascertained through the Miles Test (Mendola and Conner, 2007). Following this, a motion detection time/velocity threshold was determined for each eye separately using the custom motion salience task.

**Figure 1:**
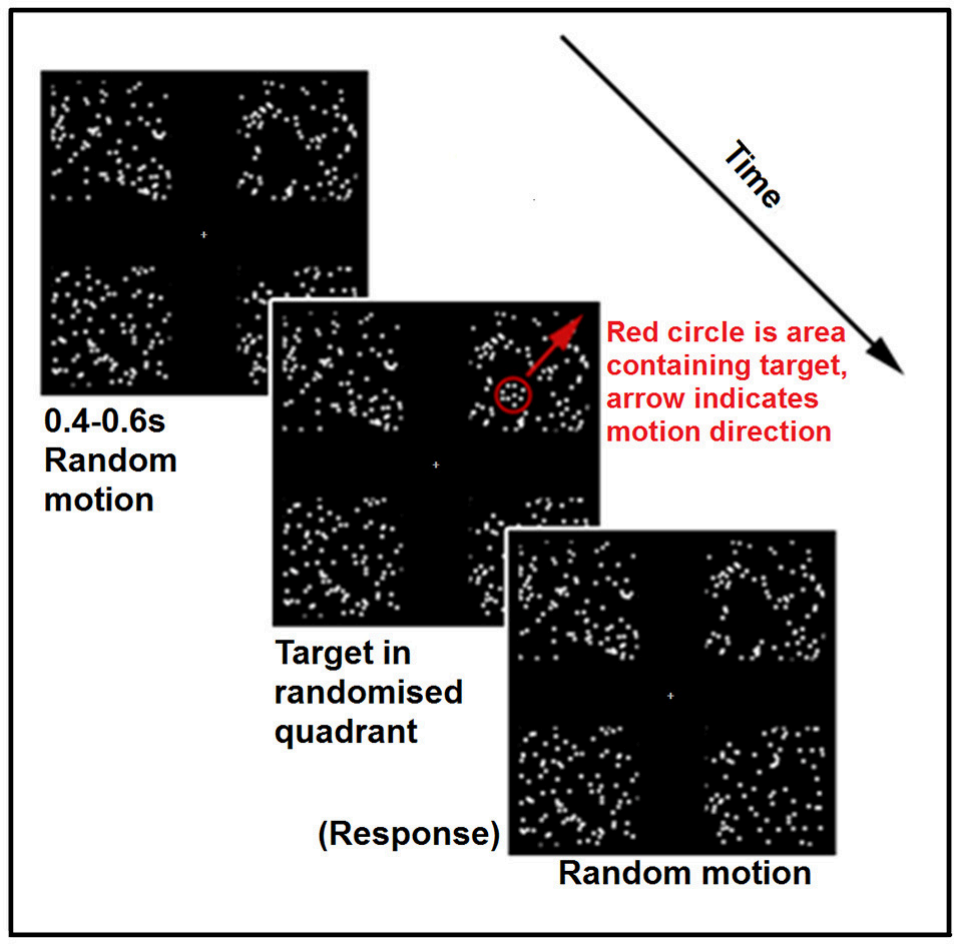
**Schematic of a single trial from the experimental condition of the Motion Salience task**. The red circle and arrow are for schematic purposes only and were not shown in the actual task.

This task was created using VPixx software (VPixx Technologies Inc., Quebec, Canada). It involved four quadrants of random dot motion positioned top left, top right, bottom left and bottom right of fixation. Each quadrant was an illusory square, 14° × 14° defined by randomly moving white dots (i.e., 0% coherence) with a density of 0.46 dots per square degree, and speed of 3°/s. Dots were white on a black background (see Figure 1) with a diameter of 0.5°, and a limited dot life time of 100 ms with replacement of each dot in a random location within the same quadrant.

The target was formed by dots within a circular region (diameter = 4°) moving with 100% coherence away from the center of the display. The circular patch was superimposed over one of the four quadrants containing randomly moving dots, such that the whole 4° circular region was in front of randomly moving dots in the background. The coherently moving dots forming the target had the same size, density and brightness as the background randomly moving dots. A gray crosshair was presented in the center of the screen throughout the whole task. Subjects were instructed to first fixate on the central crosshair and were not given further instruction with regard to eye movements. It was expected that the target when sufficiently salient would grab attention and would induce an involuntarily driven saccade to the fast central-to-peripheral movement of the target.

On each trial the target appeared randomly in one of the four quadrants, moving in a central to peripheral direction across the diagonal of the quadrant (i.e., along the diagononal of the quadrant, away from central fixation), and could be detected among the background motion on the basis of both its coherence and speed. Subjects indicated in which quadrant the target appeared via a button-press using a four-alternate forced-choice paradigm. An adaptive two-up one-down staircase procedure was used to determine the threshold for correct detection of the direction of the circular target patch of coherent dots, with chance-level performance at 25%, and threshold thus defined at the 79.1% correct level (Levitt, 1971). The initial target speed was 30°/s, with two consecutive correct responses resulting in the speed increasing by 5°/s, and a single incorrect response resulting in the speed reducing by 5°/s. It should be noted that as target speed increased, the total viewing time of the target across the constant screen distance was proportionally reduced. Thus, increased velocity resulting in reduced exposure time could reduce salience. The staircase procedure terminated after 6 reversals, with threshold calculated as the average speed at the last 4 reversals. The task was completed 3 times for each eye, with the average across the three repeats taken to be the motion threshold through this eye.

### fMRI data acquisition

#### Visual tasks

In order to assess the major neural regions involved in generating eye movements and visual attention allocation mechanisms (Bisley, 2011) we explored the activation patterns for the two tasks in order to localize the key cortical regions associated with visual and attention processing. A block design was utilized during fMRI recording with periods of task performance interspersed with rest periods.

The motion salience task was administered in the fMRI experiment as described in the behavioral experiment, except that targets were always presented with a speed of 30°/s, to ensure that the target was clearly visible through each eye for all subjects. Subjects were asked to fixate on the fixation cross and to try to determine the correct quadrant in which the target was located. Manual button responses were not required in order to exclude activations induced by finger movements. During active blocks, there were 10 trials in a row, with each trial lasting 2 s and consisting of random dot motion across the four quadrants, then the addition of a brief coherence-defined form target (lasting approximately 700 ms), with the random dot motion continuing afterwards. Baseline blocks also lasted 20 s and consisted of only the random dot motion across the four quadrants, with no targets presented. The entire task included 10 repeats of motion then baseline blocks and lasted 400 s. The task was repeated for each eye separately.

**Figure 2:**
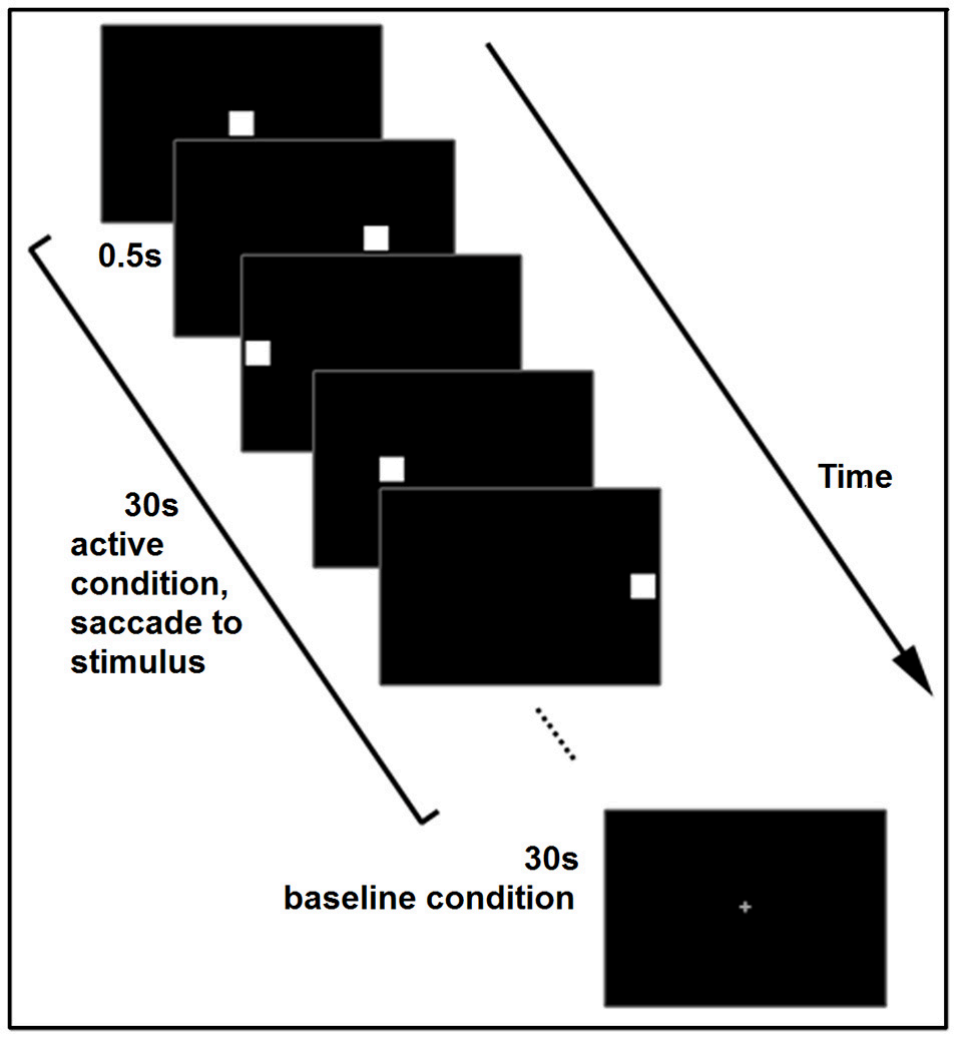
**Schematic of the voluntary saccade task, illustrating one active and one baseline block**. The task consisted of 5 blocks.

The voluntary saccade task was presented as previously described by Sestieri et al. (2007) and aimed to elicit voluntary saccade-related BOLD activity (see Figure 2). In the active condition, a white square subtending 2.18° of visual angle appeared in one of five locations: centrally, or at 6° or 12° to the left or right of center, in random order. One square appeared every 0.5 s, for a period of 30 s. The baseline condition consisted of a fixation crosshair presented for 30 s. Subjects were asked to fixate on the crosshair during baseline blocks and then to actively shift their eyes to follow the relocation of the squares during active blocks. The whole task consisted of 5 active and 5 baseline blocks, and lasted 300 s. Subjects completed the task once for each eye.

#### fMRI parameters

MRI data were collected on a 3.0 Tesla MR, 12 channel head coil scanner (Trio Tim system; Siemens, Germany). fMRI scans were performed with an echo planar imaging (EPI) sequence. A head restraint was used to ensure the subjects maintained a stable position. In the motion-salience attention task, the scan parameters were: repetition time = 2,000 ms, echo time = 30 ms, flip angle = 90°, matrix = 64 × 64, field of view = 192 × 192 mm, slice thickness = 3 mm and slice gap = 0 mm, voxel size = 3 × 3 × 3 mm. Each brain volume comprised 36 axial slices, and each functional run contained 200 volumes. Acquisition during the 2nd task was the same as during the first, except TR was 3 s and total number of volumes was 100.

A magnetization prepared rapid gradient-echo imaging (MP-RAGE) sequence was used to acquire structural T1-weighted images in a sagittal orientation. The parameters were as follows: repetition time = 2,000 ms, echo time = 2.5 ms, flip angle = 9°, matrix = 256 × 256, field of view = 256 × 256 mm, slice thickness = 1 mm and slice gap = 0 mm, voxel size = 1 × 1 × 1 mm. A total of 176 slices were acquired.

### Data processing

#### Psychophysical data processing

The average detection speed/inspection time threshold of each subject for each eye was submitted to a mixed design ANOVA, with eye as a within group factor, and participant group as a between group factor. Simple effects analyses were used to compare different levels between the independent variables.

### fMRI data preprocessing

Preprocessing was performed for individual subjects using Statistical Parametric Mapping software (SPM8, http://www.fil.ion.ucl.ac.uk/spm). Firstly, slice-timing correction was carried out, followed by realignment of images carried out to adjust for head motion during scanning. Co-registration of the functional and structural images was then performed for each subject and each image was segmented into gray and white matter, and cerebrospinal fluid, with normalization into standard Montreal Neurological Institute (MNI) space (Horn and Blankenburg, 2016). Data were spatially smoothed with a 6 mm Full Width Half Maximum kernel. Finally, SPM8 was used for first level analysis to model the data with a boxcar convolved with canonical hemodynamic response function (HRF) and to perform a general linear model regression (GLM) with a Gaussian random field family-wise error (FWE) correction (*p* < 0.05) to show the activation result of each eye in each condition.

#### ROI BOLD signal statistics

We defined 4 × 2 ROIs related to visual sensory, motion and dorsal parieto-frontal networks: V1, V5, IPS, and FEF, in the two hemispheres, by anatomical location based on standard MRI structural coordinates in MNI space. For both the V1 ROI, which was defined as Brodmann area 17 via an anatomical MRI (SPM toolbox Xjview) mask (rather than visual field retinotopy), and the V5 ROI, which was defined via a mask (8 × 8 × 8 mm cube in each hemisphere) situated around the coordinates for the centroid of motion activated voxels from previous literature (Ajina et al., 2015), there is a chance that some voxels were included that were not specifically within V1 and V5. However, Brodmann area 17 is widely equated to V1, and the motion selective complex within temporo-occipital cortex is also commonly understood to consist of V5/MT+. Hence, for simplicity we have referred to the ROIs as V1 and V5.

The IPS ROI was defined as the sulcus between superior parietal lobule and inferior parietal lobule, the anterior boundary was the post-central sulcus and the posterior boundary was the transverse occipital sulcus. Sestieri et al.'s MNI co-ordinates served as guiding information for delineating IPS (Sestieri et al., 2007). We manually built the IPS mask based on these anatomical coordinates and by selecting the gray matter within this area to be the ROI. The FEF is located around the junction of the pre-central gyrus and the middle frontal gyrus (Culham et al., 1998; Kawashima et al., 1998) and was also located for our purposes on the basis of MNI coordinates from previous human fMRI research (Luna et al., 1998; Petit and Haxby, 1999). An 8 × 8 × 8 mm area in each hemisphere was specified to be the mask for the FEF ROI. These co-ordinates are similar to those used by Secen et al. (2011).

BOLD signal change (%) was calculated using the open-source toolkit REST (available at http://www.restfmri.net).

#### ROI correlation analysis

Data from experimental and normal control subjects was binned into two groups derived from monocular stimulation of the dominant eye or the non-dominant eye (i.e., data from the amblyopic subjects' data were also separated into that derived from the non-dominant amblyopic deviating eye and the fellow dominant, non-deviating eye stimulation). Amblyopic eye-driven activation was then compared with the fellow eye and also compared with the non-dominant eye of control subjects.

Due to the fact that four of the amblyopic group subjects had left eye amblyopia and four had right eye amblyopia, BOLD data were analyzed as ipsilateral or contralateral to the amblyopic eye (rather than left/right hemisphere). Similarly, as not all control subjects had the same eye dominance, their BOLD data were analyzed as ipsilateral or contralateral to the non-dominant eye.

The correlations amongst each of the 8 ROIs of the network (ipsi- and contralateral V1, V5, IPS, and FEF) were again analyzed using the REST toolkit. The BOLD signal values of each TR in active blocks were subjected to correlation analysis between pairs of ROIs. For further statistical analysis, a Fisher r-to-z transformation was performed to improve the normality of the correlation coefficients (Yuan et al., 2013). For each network connection, a separate 2 (dominant eye, non-dominant eye) by 2 (subject group) mixed design ANOVA was used to determine the strength of the correlation, with simple effect analyses used in the event of significant interactions.

## Results

### Psychophysics

**Figure 3:**
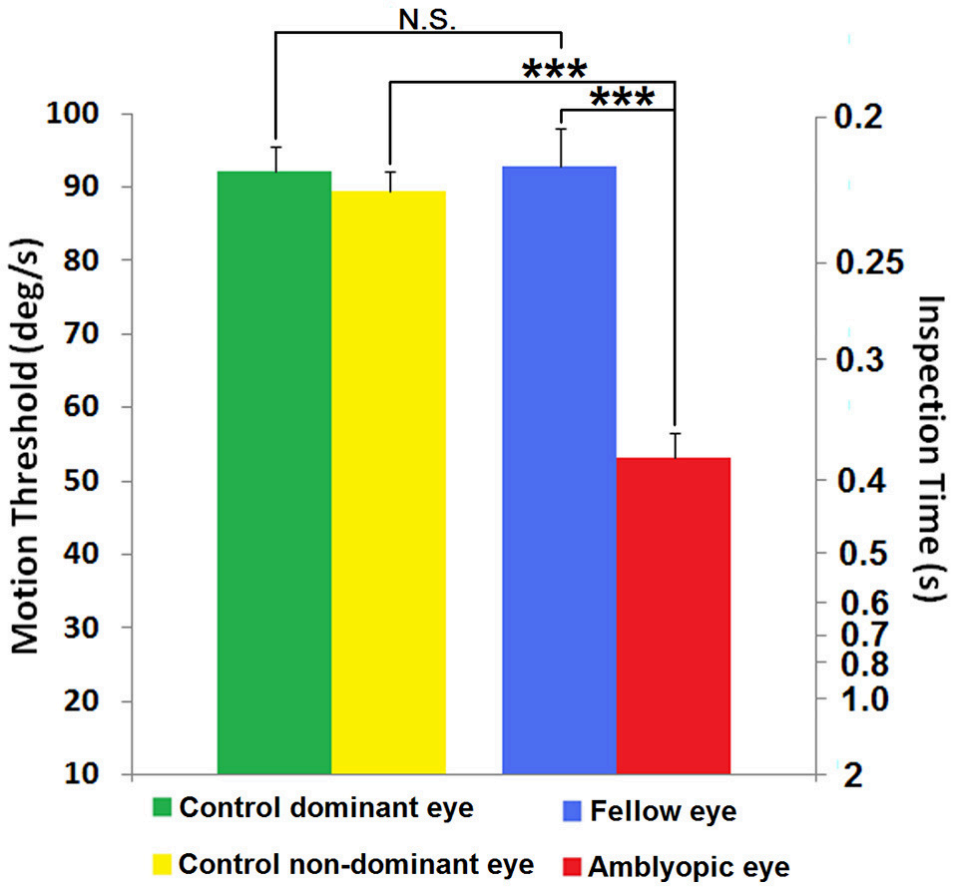
**Threshold performance on the motion salience attention task**. Data are shown for each eye group as mean target velocity thresholds (left axis) and the corresponding mean target detection inspection time (right axis) calculated using the corresponding speed from the left axis. Green and yellow bars represent the mean thresholds of the dominant and non-dominant eyes of the controls (*N* = 8). Blue and red bars represent the mean threshold of the fellow and amblyopic eyes respectively of the group of *N* = 8 strabismics with amblyopia. Note that higher thresholds for speed of coherently moving dots indicate better performance. Error bars represent 1 SEM. N.S., non-significant (*p* > 0.05); ^***^p < 0.001.

The 2 × 2 mixed design ANOVA on motion threshold performance revealed a main effect of eye, *F*_(1, 14)_ = 64.84, *p* < 0.001, $\eta_{p}^{2}$ = 0.822; a main effect of group, *F*_(1, 14)_ = 14.54, *p* = 0.002, $\eta_{p}^{2}$ = 0.510; and a significant interaction between eye and group, *F*_(1, 14)_ = 49.42, *p* < 0.001, $\eta_{p}^{2}$ = 0.779. Simple main effects analyses showed that amblyopic eye motion thresholds (visual movement in degrees°/s) were significantly slower than non-amblyopic fellow eye thresholds (*p* < 0.001) (see Figures S1, S2 showing each participant's individual staircase sequences). Similarly when motion thresholds of the amblyopic eye were compared with the motion thresholds of the non-dominant eye of control subjects, the amblyopic eye required slower target velocities to acquire the target (*p* < 0.001). Because the target makes a linear transit of the background random dot patch, higher velocity mandates shorter exposure. Hence the motion velocity threshold result could also be viewed as an Inspection Time measure (right axis of Figure 3). The fellow eye of the 8 amblyopes showed no difference in performance from the dominant eye of the control subjects (*p* = 0.920), whilst no difference between eyes of control participants was seen (*p* = 0.482). The results for each eye of the amblyopic subjects and control subjects are shown in Figure 3, where it can be seen that amblyopes' threshold performance averaged 53.60°/s (*SD* = 9.24). It should be noted that although threshold velocity was significantly reduced for amblyopes, the adaptive 2-up 1-down procedure ensured all participants were performing the task at threshold with percent correct close to the theoretical 79.1% level of the psychometric function.

The average speed threshold for the amblyopic eye was significantly slower than that for the non-amblyopic fellow eye in the amblyopes and also slower than dominant and non-dominant eye viewing in normal controls, indicating poorer motion-driven attention performance through the amblyopic eye. By comparison, average speed threshold for the fellow eyes was not significantly different from that of the dominant eyes (for the normal subject group), indicating the motion-driven attention function of fellow eyes is normal. There was also no significant difference of speed threshold between dominant and non-dominant eye of control.

### Task related fMRI activation

To ensure that all participants in the first fMRI experiment could correctly detect the rapidly moving motion salience target, a target speed of 30°/s—slower than the speed threshold through any eye of all participants—was employed.

**Figure 4:**
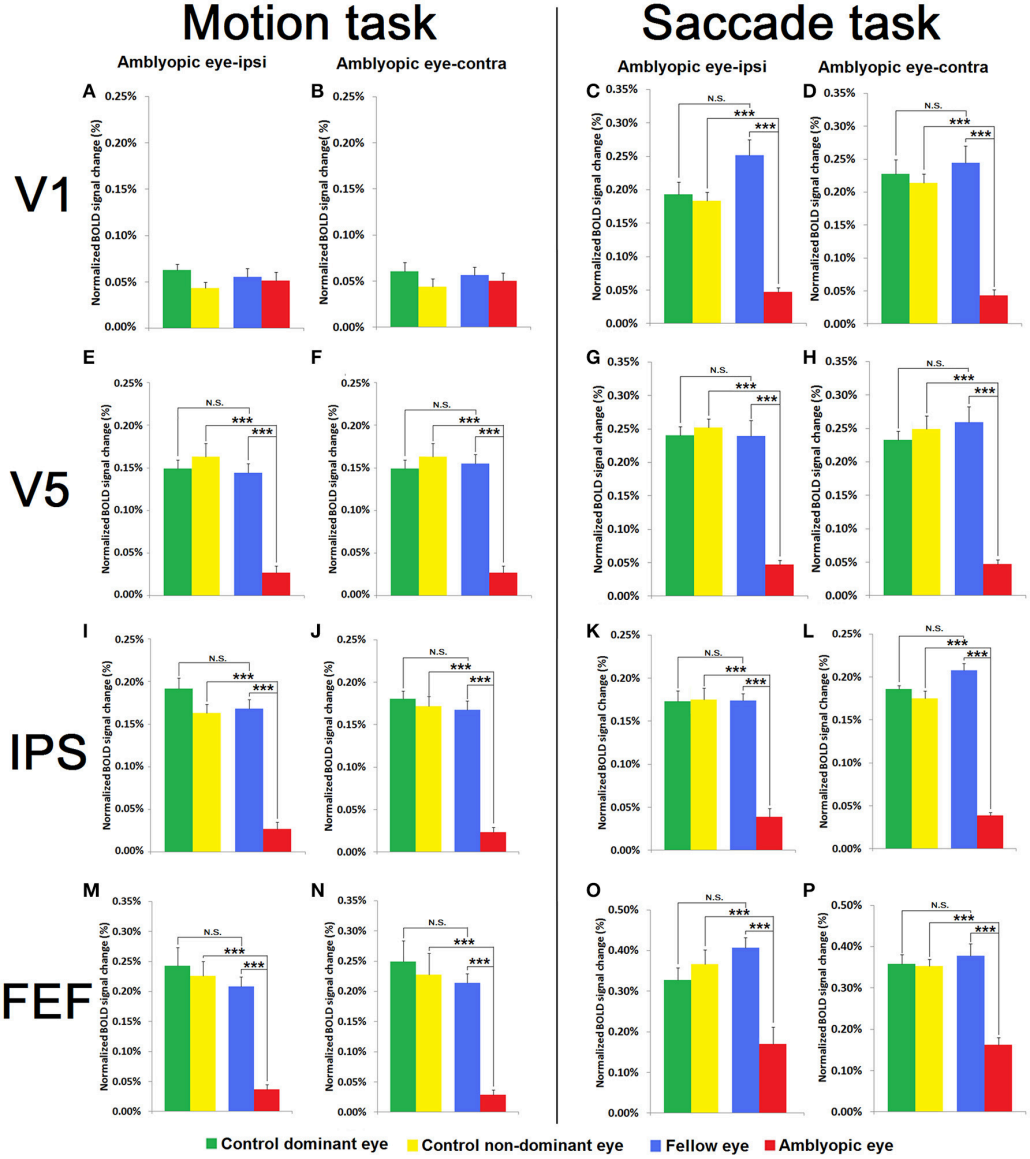
**Comparison between Motion Salience and Saccade Tasks of BOLD activation in V1 (A–D), V5 (E–H), IPS (I–L), and FEF(M–P) for both visual tasks in both hemispheres relative to the non-dominant and dominant eyes of amblyopic subjects and healthy control participants**. Data are shown as BOLD change (%) for each eye of the normal controls (*N* = 8) [Green and yellow bars represent the mean BOLD change (%) of the dominant and non-dominant eyes of the controls, respectively] and for the group of strabismic amblyopes (*N* = 8) [Blue and red bars represent the mean BOLD change (%) of the fellow and amblyopic eyes, respectively] in both motion salience and saccade tasks. ^***^ *p* < 0.001; N.S., not significant.

Reduced activation was observed for viewing through the Amblyopic eye compared with Fellow eye and with either eye group of the Controls for all ROI's except V1, during both the Motion and the Saccade tasks (see Figure 4). The reduced activation in V1 for all eye groups in the motion task, compared with activation in the other ROIs, is likely to be associated with the small relative differences in mean contrast of baseline background and target stimuli during this task rather than a unique reflection of ocularly induced activation. For example, in the preferentially stimulus-driven random-dot motion coherence task, baseline to target task would be expected to show little relative change in mean contrast or activation between eye groups, as the total number of moving dots remain the same throughout, with only the percentage of coherent dots changing. By comparison, in the goal-directed voluntary saccade task, the baseline to target activation is a shift from a small white central fixation cross on a black background to a white square subtending 2.18° of visual angle, appearing abruptly and differing substantially in mean overall luminance and contrast.

#### Motion salience task

As shown in Figure 4, the BOLD activation in V1 for the motion salience task was low in all eye groups when compared with V5, IPS, and FEF. A 3-way mixed design ANOVA (eye × hemisphere × group) for V1 activation on the motion task with eye (dominant, non-dominant) and hemisphere (contralateral, ipsilateral to the viewing eye) as within subject factors, and group (amblyopia, control) as between subject factors, showed no significant interaction effects. The only significant result was a main effect for eye, *F*_(1, 14)_ = 5.01, *p* = 0.042, $\eta_{p}^{2}$ = 0.264 indicating greater BOLD response for dominant/fellow eye compared with non-dominant/amblyopic eye viewing.

ANOVA for V5 activation in the motion task showed a different pattern of results to that seen in V1. Main effects were found for eye and also for group, though not hemisphere. Additionally a 2-way interaction effect was observed between eye and group [*F*_(1, 14)_ = 139.34, *p* < 0.001, $\eta_{p}^{2}$ = 0.91]. Subsequent simple main effects analysis showed that amblyopic eye viewing resulted in reduced V5 activation when compared with fellow eye viewing (*p* < 0.001, $\eta_{p}^{2}$ = 0.94), and when compared with V5 activation for control non-dominant eye viewing (*p* < 0.001, $\eta_{p}^{2}$ = 0.93). Amblyopes' fellow eye viewing did not differ from control participants' dominant eye in V5 activation (*p* = 0.887). No 3-way interaction effect was established.

For IPS, ANOVA on the motion task showed a similar pattern of results as for V5 analyses. Main effects of eye and group were established, along with a significant 2-way interaction between eye and group [*F*_(1, 14)_ = 29.25, *p* < 0.001, $\eta_{p}^{2}$ = 0.68]. Simple effects analyses showed that there was an eye difference in the amblyopic group, with reduced BOLD activation in the amblyopic eye (*p* < 0.001, $\eta_{p}^{2}$ = 0.90), and also showing reduced activation comparing amblyopic eye with control participant non-dominant eye conditions (*p* < 0.001, $\eta_{p}^{2}$ = 0.84). No eye difference in the control group (*p* = 0.326, $\eta_{p}^{2}$ = 0.07) nor between fellow and control dominant eye (*p* = 0.435, $\eta_{p}^{2}$ = 0.04) activation was established. No other main effects or interactions were established.

Finally, a similar pattern of activation results was again seen in FEF for the motion task (see Figure 4). The BOLD activation in FEF during amblyopic eye viewing was much lower than activation through the fellow eye and through the dominant and also the non-dominant eye viewing responses of control subjects. A 3-way mixed design ANOVA showed main effects of eye and group. A significant interaction was observed between eye and group [*F*_(1, 14)_ = 23.88, *p* < 0.001, $\eta_{p}^{2}$ = 0.63]. Simple main effects analysis showed that amblyopic eye viewing produced significantly reduced FEF activation compared with fellow eye viewing [*p* < 0.001, $\eta_{p}^{2}$ = 0.82), and also when compared with non-dominant eye viewing of control subjects (*p* < 0.001, $\eta_{p}^{2}$ = 0.86). Amblyopic group fellow eye viewing did not result in significantly different FEF activation when compared with control group dominant eye viewing (*p* = 0.192, $\eta_{p}^{2}$ = 0.12). No other main effects or interaction effects were established.

#### Saccade task

In the saccade task, ANOVA analysis of V1 activation established main effects of eye and also of group. A significant 2-way interaction between eye and hemisphere was established *F*_(1, 14)_ = 5.40, *p* = 0.036, $\eta_{p}^{2}$ = 0.28, though this was not explored further as it did not involve the relevant group factor. A 2-way interaction was found between eye and group, *F*_(1, 14)_ = 40.08, *p* < 0.001, $\eta_{p}^{2}$ = 0.74. Simple effects analysis showed that V1 activation following amblyopic eye viewing was reduced compared with fellow eye viewing (*p* < 0.001, $\eta_{p}^{2}$ = 0.87), as well as when compared with control group non-dominant eye viewing (*p* < 0.001, $\eta_{p}^{2}$ = 0.91). Fellow eye and control participant dominant eye (*p* = 0.147, $\eta_{p}^{2}$ = 0.14), as well as control dominant and non-dominant eye (*p* = 0.605, $\eta_{p}^{2}$ = 0.02) comparisons did not differ in V1 activation. No other main effect or interaction effects were significant.

In the Saccade task, V5 activation showed the same pattern of results, showing a 2-way interaction between eye and group [*F*_(1, 14)_ = 74.68, *p* < 0.001, $\eta_{p}^{2}$ = 0.84]. Simple effects analysis showed that amblyopic eye viewing resulted in reduced V5 activation when compared with fellow viewing (*p* < 0.001, $\eta_{p}^{2}$ = 0.91), and when compared with V5 activation for control non-dominant eye viewing (*p* < 0.001, $\eta_{p}^{2}$ = 0.95). However, no differences were established between fellow eye and control dominant eye (*p* = 0.705, $\eta_{p}^{2}$ = 0.01), and also between control dominant and non-dominant eyes (*p* = 0.555, $\eta_{p}^{2}$ = 0.03). No other main effect or interaction effects were significant.

ANOVA for IPS on the saccade task revealed significant main effects of eye and hemisphere along with a 2-way interaction between eye and hemisphere. In addition a 2-way interaction between eye and group [*F*_(1, 14)_ = 81.51, *p* < 0.001, $\eta_{p}^{2}$ = 0.85] was established. Simple effects analyses indicated that amblyopes showed reduced activation following stimulation through the amblyopic compared with fellow eye viewing (*p* < 0.001, $\eta_{p}^{2}$ = 0.93) and also compared with control non-dominant eye viewing (*p* < 0.001, $\eta_{p}^{2}$ = 0.91). Fellow eye and control participant dominant eye (*p* = 0.204, $\eta_{p}^{2}$ = 0.11), as well as control dominant and non-dominant eye (*p* = 0.732, $\eta_{p}^{2}$ = 0.01) comparisons did not differ in V1 activation. No other main effect or interaction effects were significant.

Finally, ANOVA for FEF on the saccade task established a main effect of group and of eye, along with a 2-way interaction between eye and group [*F*_(1, 14)_ = 41.05, *p* < 0.001, $\eta_{p}^{2}$ = 0.75] with no other 2-way interactions. Simple effects analyses to interpret this interaction found that amblyopic eye compared with fellow eye viewing produced significantly reduced FEF activation (*p* < 0.001, $\eta_{p}^{2}$ = 0.84), and also when compared with non-dominant eye viewing from control subjects (*p* = 0.001, $\eta_{p}^{2}$ = 0.76). FEF activation for the amblyopic group fellow eye viewing did not differ from control group dominant eye viewing (*p* = 0.100, $\eta_{p}^{2}$ = 0.18), with no difference also found between control dominant and non-dominant eye viewing activations (*p* = 0.547, $\eta_{p}^{2}$ = 0.03).

### Functional connectivity of attention network regions—correlational analysis

While it is clear that stimulation through the amblyopic eye shows lower percent BOLD change than through the fellow eye or control eyes, we were interested in how functionally correlated the attention networks formed by the eight ROIs (ipsilateral/contralateral V1, V5, IPS, and FEF) were when stimulated through each of the four viewing conditions. From the eight selected ROIs, there are 28 possible functional connections characterizing the network. In each task we extracted the BOLD signal for each ROI in the active blocks of the given task and then calculated the correlation coefficient for each pairing of ROIs. A higher correlation coefficient between two ROIs would be representative of greater functional connection.

Given the large number of possible functional connections, and consequently the large number of ANOVA, we present these results showing main effect of eye, main effect of group, and the interaction effect in full in the Supplementary Information (Table S1: Motion task results; Table S2: Saccade task results). In the main text below we focus on the significant group by eye interactions (setting *alpha* = 0.0036 after making a Bonferroni correction for the large number of comparisons), as these describe the hypothesized connectivity impairments in amblyopic eye viewing conditions.

#### Motion task

Across all possible network pairs, there were no significant main effects of group. There was only a single instance of a main effect of eye, for the iFEF (FEF ipsilateral to the non-dominant eye) to cFEF (FEF contralateral to the non-dominant eye) connection, indicating that across both groups, this functional connection was stronger for dominant (or fellow) eye compared with non-dominant (or amblyopic) eye viewing. Significant group by eye interactions were found for the iFEF-cV1 and for the cFEF-iV5 connections. No other interaction effects from amongst the network connections were significant at the 0.0036 level. As is seen in Table 2, simple effects analyses reveal that these interactions were driven by reduced correlated activity from amblyopic eye viewing compared with fellow eye viewing (and also for the iFEF-cV1 connection, when compared with control participants' non-dominant eye viewing). Fellow eye viewing did not significantly differ from control participant dominant eye viewing conditions.

**Table 2:** **Simple main effects analyses for significant Eye × Group Interactions for the motion task**.

	**Amblyopic vs. fellow eye**	**Control non-dominant vs. dominant eye**	**Amblyopic vs. control non-dominant eye**	**Fellow vs. control dominant eye**
iFEF – cV1	*p* = 0.002	*p* = 0.479	*p* = 0.010	*p* = 0.622
cFEF – iV5	*p* = 0.001	*p* = 0.330	*p* = 0.110	*p* = 0.072^∧^

Simple main effects are shown for the only two Eye × Group interactions that were significant (for alpha = 0.0036).

∧ *Indicates Amblyopes had a statistically significantly higher average correlation than Control participants for their respective dominant eye comparisons*.

**Figure 5:**
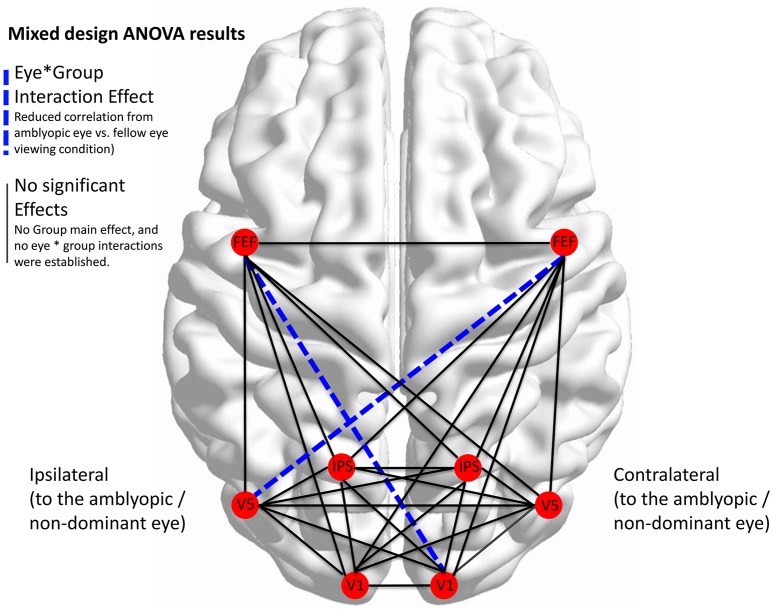
**Disturbed network functional connectivity for the Motion Salience task**. Functional connectivity correlations for all 8 ROIs (i.e., V1, V5, IPS, and FEFs ipsi- and contra- to the non-dominant eye of amblyopes and controls). Dashed blue lines denote a significant eye by group interaction effect, with simple effects analyses indicating reduced correlations for that connection from amblyopic eye viewing compared with fellow eye viewing. Statistics corrected for multiple comparison (alpha = 0.0036).

Figure 5 below shows the combination of all 28 functional connections for the Motion Salience task, and highlights the disturbed connections in amblyopic subjects. Note that for comparison purposes all the activation maps have been drawn showing the left eye as the non-dominant eye (i.e., as the amblyopic eye for the Amblyopia group and as the non-dominant eye for the Controls).

#### Saccade task

A similar set of correlation pairings was carried out for the voluntary saccade task. However, results from all sets of mixed design ANOVA's failed to reveal any main effects (eye or group), nor any significant eye*group interaction effects, with alpha = 0.0036. It should be noted that although the iV1-cV1 connection revealed a marginally significant interaction effect (*p* = 0.004), simple effects analyses demonstrated that this interaction was driven by the surprising significantly higher correlations produced by control non-dominant-eye compared with dominant eye viewing (*p* = 0.014), whereas strabismic participants with amblyopia showed marginally lower correlations from amblyopic eye compared with fellow eye viewing conditions (*p* = 0.051).

## Discussion

In this study, we have demonstrated psychophysically that adult strabismic amblyopic eyes show deficits in threshold velocity (and exposure time) needed to correctly detect movement of a group of coherently moving dots requiring induced visual attention. Our fMRI exploration of the neural mechanisms underlying this behavioral motion-driven attention deficit, also demonstrated corresponding BOLD activation differences in key nodes of the cortical visual attention network (V1, V5, IPS, and FEF) (Corbetta and Shulman, 2002) using our two tasks that tapped into stimulus-driven motion salience attention and goal-directed voluntary saccade generated attention shifts (Corbetta, 1998; Vernet et al., 2014). In particular, statistically reduced BOLD change was established for all areas of the attention network except V1, for both tasks when driven through the amblyopic eye (See Figure 4). Saccade task induced V1 activation was also statistically less through the amblyopic eye, whereas all motion salience task V1 activation, was minimal under all four monocular conditions. For the motion salience task the relatively low V1 activation likely reflects the difference between conditions with and without a target—each stimulus has four regions with random motion, and potentially V1 is not responding selectively to the coherent dots. In V5, by comparison, the increase in differential signal to the motion salience task points to the coding of the target as a foreground object of interest (Bullier, 2001). Interestingly, examination of functional connectivity within the parieto-frontal visual attention network, comprising the four ROIs (left/right IPS, FEF), revealed a significant functional disconnection of the FEF during the motion salience task, but not during the goal-directed voluntary saccadic task. In particular, analyses of functional connectivity between the V1, V5, IPS, and FEF of the two hemispheres indicated significantly less connectivity between bilateral FEF, V1, and V5 during the motion salience task when viewing though the amblyopic eye.

Pronounced psychophysical impairments when viewing through the amblyopic eye were not unexpected, given previously reported deficits in attention and motion processing (Asper et al., 2000a; Hayward et al., 2011; Thompson et al., 2012; Meier et al., 2016). However, our finding of normal motion thresholds (velocity) through the fellow non-deviating eye is perhaps unexpected given reports of abnormal motion-from-form perception through the fellow eye (Giaschi et al., 1992; Ho et al., 2005). On the other hand Ho et al. (2005) have previously suggested that there may be a difference in this regard between participants with anisometropia and strabismic amblyopia as used here. Thompson et al. (2012) have also noted differences in patterns of activation to different aspects of motion (i.e., coherent vs non-coherent plaids) that may apply to our psychophysical stimuli that required detection in order to discriminate motion direction. Low attentional temporal sampling (Landau and Fries, 2012) ability through the amblyopic eye, when considering the motion inspection time differences in the current results (see Figure 4), is a possible area for exploration in future research.

It has been previously noted that strabismic amblyopes counted groups of objects surprisingly inaccurately and that this inaccuracy could not be accounted for by low level considerations such as blur, visibility, crowding, under-sampling or topographical jitter (Sharma et al., 2000). Indeed, Sharma et al. (2000) commented that such counting deficits reflected a “higher-level limitation in the number of features the amblyopic visual system can individuate.”

fMRI studies of amblyopia indicate that BOLD activation of ocular dominance columns in V1 is strongly biased toward the non-amblyopic eye, whilst the columns driven by the amblyopic eye are dramatically decreased in size (Goodyear et al., 2002; Liu et al., 2004). The non-amblyopic eye drives most of the neural networks and visual functions (Li et al., 2011; Secen et al., 2011), although normal processing of moving plaid patterns by the amblyopic eye is associated with activation differences within hMT+ as well as pulvinar and Area 3A (Thompson et al., 2012). While Secen et al. (2011) showed impairments in frontal and parietal regions when viewing through the amblyopic eye, the use of a single bilateral ROI for each region did not facilitate comment on the possible lateralization of these impairments, as highlighted in the effective connectivity analyses in the present findings. The current study however has established that for motion-driven attention there are functional connectivity abnormalities associated with bilateral FEF from amblyopic eye viewing, whereas there were no impaired connections from the saccade task.

Attentional deficits associated with amblyopia have often been proposed, as it has long been recognized clinically (Schor, 1975) that the time taken by the amblyopic eye to assume fixation is longer than the time to fixation of the fellow eye or control eyes. Thiel and Sireteanu (2009) found that subjects with strabismic amblyopia showed a bilateral rightward bias in a line bisection task, reminiscent of classic attentional neglect. Our psychophysical results also indicate that the activation of motion-driven attention in untrained adult strabismic amblyopes is less when using the amblyopic eye rather than their fellow eye, or either dominant of non-dominant eye of control subjects. The reductions in BOLD activation, localized in V5, IPS, and FEF, were established across both conditions requiring attention to detect quadrant of the moving object and also when making deliberate saccadic eye movements to the transient appearance of the white square target.

Goal directed visual attention is a neural function critically associated with eye movements and the control of visual information processing, while contributing to perception of the external world (Corbetta and Shulman, 2002). Eye movements are strongly related to shifts in visual attention, with peripheral fluctuation in scene conditions automatically drawing attention via saccadic eye movements (Bruce and Goldberg, 1985; Schiller et al., 1987). In addition, consciously-driven eye movements allow objects of interest to be inspected for certain aspects or details. When functioning normally, the visual neural system must maintain a subtle balance between eye movement and fixation. However, as alluded to above (Schor, 1975), anomalous fixation and abnormal eye movement function by the amblyopic eye throughout early development and childhood (Crewther, 2002), could be predicted to affect maturation of visual attention (Singer, 1982a).

In conclusion, the slower motion thresholds recorded through the amblyopic eye indicate a need for longer exposure to the moving target in order to be perceived through the amblyopic eye. Similarly BOLD activations during voluntary goal-directed saccades and motion salience-driven eye movements performed by the amblyopic eye were significantly lower than that generated through the fellow eye or for the eyes of normal subjects. Thus, our results indicate that strabismic amblyopia is associated with a generalized reduction in activation across the attention network for stimulation through the amblyopic eye. In addition, a specific deficit in functional connectivity with the frontal eye fields in both hemispheres and the visual attentional networks when viewing through the strabismic eye was found to be associated with motion salience but not goal-directed voluntary saccades.

## Ethics statement

This study was carried out in accordance with the recommendations of the Declaration of Helsinki. The Human Ethics Committees of La Trobe University, Melbourne, Australia; and Southwest Hospital, Chongqing, China approved the protocol. All subjects gave written informed consent.

## Author contributions

HW: Research design, subject collection, data collection, data analysis, manuscript writing. SC: Research design, manuscript writing. ML: Data collection. RL: Research design, data analysis, manuscript writing. TY: Subject collection. BA: Research design. DC: Research design, data analysis, manuscript writing. JW: Data collection. ZY: Research guidance.

### Conflict of interest statement

The authors declare that the research was conducted in the absence of any commercial or financial relationships that could be construed as a potential conflict of interest. The reviewer AC and handling Editor declared their shared affiliation, and the handling Editor states that the process nevertheless met the standards of a fair and objective review.
