# Supplementary material for: Impaired Activation of Visual Attention Network for Motion Salience Is Accompanied by Reduced Functional Connectivity between Frontal Eye Fields and Visual Cortex in Strabismic Amblyopia
Source: Front Hum Neurosci. 2017 Apr 21;11:195. doi: 10.3389/fnhum.2017.00195 (PMC5399630; doi:10.3389/fnhum.2017.00195)
Supplement: Supplementary file 1 [file Table1.docx]

**Table S1: Motion Task correlation connectivity ANOVA results**

| **Connection** | **Main Effect: Eye** | **Main Effect Group** | **Eye * Group Interaction** |
| --- | --- | --- | --- |
| iV5 – cV5 | *p* = .141,  = 0.148 | *p* = .782,  = 0.006 | *p* = .243,  = 0.096 |
| iV5 – iV1 | *p* = .996,  = 0.000 | *p* = .082,  = 0.201 | *p* = .873,  = 0.002 |
| iV5 – cV1 | *p* = .852,  = 0.003 | *p* = .093,  = 0.189 | *p* = .863,  = 0.002 |
| cV5 – iV1 | *p* = .911,  = 0.001 | *p* = .506,  = 0.032 | *p* = .634,  = 0.017 |
| cV5 – cV1 | *p* = .630,  = 0.017 | *p* = .673,  = 0.013 | *p* = .675,  = 0.013 |
| iV1 – cV1 | *p* = .008,  = 0.403 | *p* = .423,  = 0.046 | *p* = .480,  = 0.036 |
| iIPS – cIPS | *p* = .423,  = 0.046 | *p* = .095,  = 0.186 | *p* = .792,  = 0.005 |
| iIPS – iV1 | *p* = .100,  = 0.182 | *p* = .745,  = 0.008 | *p* = .572,  = 0.023 |
| iIPS – cV1 | *p* = .089,  = 0.193 | *p* = .637,  = 0.016 | *p* = .707,  = 0.010 |
| cIPS – iV1 | *p* = .284,  = 0.081 | *p* = .404,  = 0.050 | *p* = .338,  = 0.065 |
| cIPS – cV1 | *p* = .065,  = 0.222 | *p* = .744,  = 0.008 | *p* = .746,  = 0.008 |
| iFEF – cFEF | *p* = .001,  = 0.530 | *p* = .897,  = 0.001 | *p* = .182,  = 0.123 |
| iFEF– iV1 | *p* = .071,  = 0.215 | *p* = .519,  = 0.030 | *p* = .073,  = 0.212 |
| iFEF– cV1 | *p* = .045,  = 0.257 | *p* = .208,  = 0.111 | *p* = .006,  = 0.427 |
| cFEF – iV1 | *p* = .008,  = 0.404 | *p* = .307,  = 0.074 | *p* = .019,  = 0.333 |
| cFEF – cV1 | *p* = .043,  = 0.261 | *p* = .143,  = 0.146 | *p* = .013,  = 0.367 |
| iIPS – iV5 | *p* = .461,  = 0.039 | *p* = .834,  = 0.003 | *p* = .171,  = 0.130 |
| iIPS – cV5 | *p* = .864,  = 0.002 | *p* = .782,  = 0.006 | *p* = .882,  = 0.002 |
| cIPS – iV5 | *p* = .363,  = 0.059 | *p* = .999,  = 0.000 | *p* = .335,  = 0.066 |
| cIPS – cV5 | *p* = .899,  = 0.001 | *p* = .628,  = 0.017 | *p* = .557,  = 0.025 |
| iFEF – iV5 | *p* = .197,  = 0.116 | *p* = .687,  = 0.012 | *p* = .014,  = 0.358 |
| iFEF – cV5 | *p* = .054,  = 0.239 | *p* = .776,  = 0.006 | *p* = .077,  = 0.206 |
| cFEF – iV5 | *p* = .053,  = 0.242 | *p* = .774,  = 0.006 | *p* = .003,  = 0.473 |
| cFEF – cV5 | *p* = .877,  = 0.002 | *p* = .554,  = 0.026 | *p* = .705,  = 0.011 |
| iFEF – iIPS | *p* = .026,  = 0.307 | *p* = .999,  = 0.000 | *p* = .143,  = 0.147 |
| iFEF – cIPS | *p* = .019,  = 0.332 | *p* = .930,  = 0.001 | *p* = .033,  = 0.286 |
| cFEF – iIPS | *p* = .651,  = 0.015 | *p* = .601,  = 0.020 | *p* = .890,  = 0.001 |
| cFEF – cIPS | *p* = .984,  = 0.000 | *p* = .867,  = 0.002 | *p* = .667,  = 0.014 |
