# Supplementary material for: Impaired Activation of Visual Attention Network for Motion Salience Is Accompanied by Reduced Functional Connectivity between Frontal Eye Fields and Visual Cortex in Strabismic Amblyopia
Source: Front Hum Neurosci. 2017 Apr 21;11:195. doi: 10.3389/fnhum.2017.00195 (PMC5399630; doi:10.3389/fnhum.2017.00195)
Supplement: Supplementary file 2 [file Table2.docx]

**Table S2: Saccade Task correlation connectivity ANOVA results**

| **Connection** | **Main Effect: Eye** | **Main Effect Group** | **Eye * Group Interaction** |
| --- | --- | --- | --- |
| iV5 – cV5 | *p* = .533,  = 0.028 | *p* = .039,  = 0.270 | *p* = .963,  = 0.000 |
| iV5 – iV1 | *p* = .474,  = 0.037 | *p* = .591,  = 0.021 | *p* = .683,  = 0.012 |
| iV5 – cV1 | *p* = .227,  = 0.102 | *p* = .372,  = 0.057 | *p* = .958,  = 0.000 |
| cV5 – iV1 | *p* = .336,  = 0.066 | *p* = .959,  = 0.000 | *p* = .274,  = 0.085 |
| cV5 – cV1 | *p* = .135,  = 0.153 | *p* = .290,  = 0.079 | *p* = .571,  = 0.023 |
| iV1 – cV1 | *p* = .639,  = 0.016 | *p* = .714,  = 0.010 | *p* = .004,  = 0.446 |
| iIPS – cIPS | *p* = .953,  = 0.000 | *p* = .166,  = 0.132 | *p* = .331,  = 0.067 |
| iIPS – iV1 | *p* = .445,  = 0.042 | *p* = .769,  = 0.006 | *p* = .710,  = 0.010 |
| iIPS – cV1 | *p* = .462,  = 0.039 | *p* = .191,  = 0.119 | *p* = .869,  = 0.002 |
| cIPS – iV1 | *p* = .754,  = 0.007 | *p* = .995,  = 0.000 | *p* = .729,  = 0.009 |
| cIPS – cV1 | *p* = .557,  = 0.025 | *p* = .472,  = 0.038 | *p* = .904,  = 0.001 |
| iFEF – cFEF | *p* = .417,  = 0.048 | *p* = .561,  = 0.025 | *p* = .295,  = 0.078 |
| iFEF– iV1 | *p* = .345,  = 0.064 | *p* = .191,  = 0.119 | *p* = .606,  = 0.019 |
| iFEF– cV1 | *p* = .709,  = 0.010 | *p* = .645,  = 0.016 | *p* = .314,  = 0.072 |
| cFEF – iV1 | *p* = .738,  = 0.008 | *p* = .431,  = 0.045 | *p* = .326,  = 0.069 |
| cFEF – cV1 | *p* = .789,  = 0.005 | *p* = .252,  = 0.093 | *p* = .802,  = 0.005 |
| iIPS – iV5 | *p* = .359,  = 0.060 | *p* = .703,  = 0.011 | *p* = .921,  = 0.001 |
| iIPS – cV5 | *p* = .191,  = 0.119 | *p* = .441,  = 0.043 | *p* = .728,  = 0.009 |
| cIPS – iV5 | *p* = .914,  = 0.001 | *p* = .677,  = 0.013 | *p* = .566,  = 0.024 |
| cIPS – cV5 | *p* = .674,  = 0.013 | *p* = .379,  = 0.056 | *p* = .763,  = 0.007 |
| iFEF– iV5 | *p* = .809,  = 0.004 | *p* = .392,  = 0.053 | *p* = .360,  = 0.060 |
| iFEF– cV5 | *p* = .341,  = 0.065 | *p* = .167,  = 0.132 | *p* = .514,  = 0.031 |
| cFEF – iV5 | *p* = .695,  = 0.011 | *p* = .896,  = 0.001 | *p* = .790,  = 0.005 |
| cFEF – cV5 | *p* = .585,  = 0.022 | *p* = .154,  = 0.139 | *p* = .779,  = 0.006 |
| iFEF – iIPS | *p* = .809,  = 0.004 | *p* = .975,  = 0.000 | *p* = .935,  = 0.000 |
| iFEF – cIPS | *p* = .299,  = 0.077 | *p* = .989,  = 0.000 | *p* = .218,  = 0.106 |
| cFEF – iIPS | *p* = .267,  = 0.087 | *p* = .664,  = 0.014 | *p* = .978,  = 0.000 |
| cFEF – cIPS | *p* = .956,  = 0.000 | *p* = .395,  = 0.052 | *p* = .576,  = 0.023 |
