# Supplementary material for: Impaired Activation of Visual Attention Network for Motion Salience Is Accompanied by Reduced Functional Connectivity between Frontal Eye Fields and Visual Cortex in Strabismic Amblyopia
Source: Front Hum Neurosci. 2017 Apr 21;11:195. doi: 10.3389/fnhum.2017.00195 (PMC5399630; doi:10.3389/fnhum.2017.00195)
Supplement: Supplementary file 7 [file Image1.PDF]

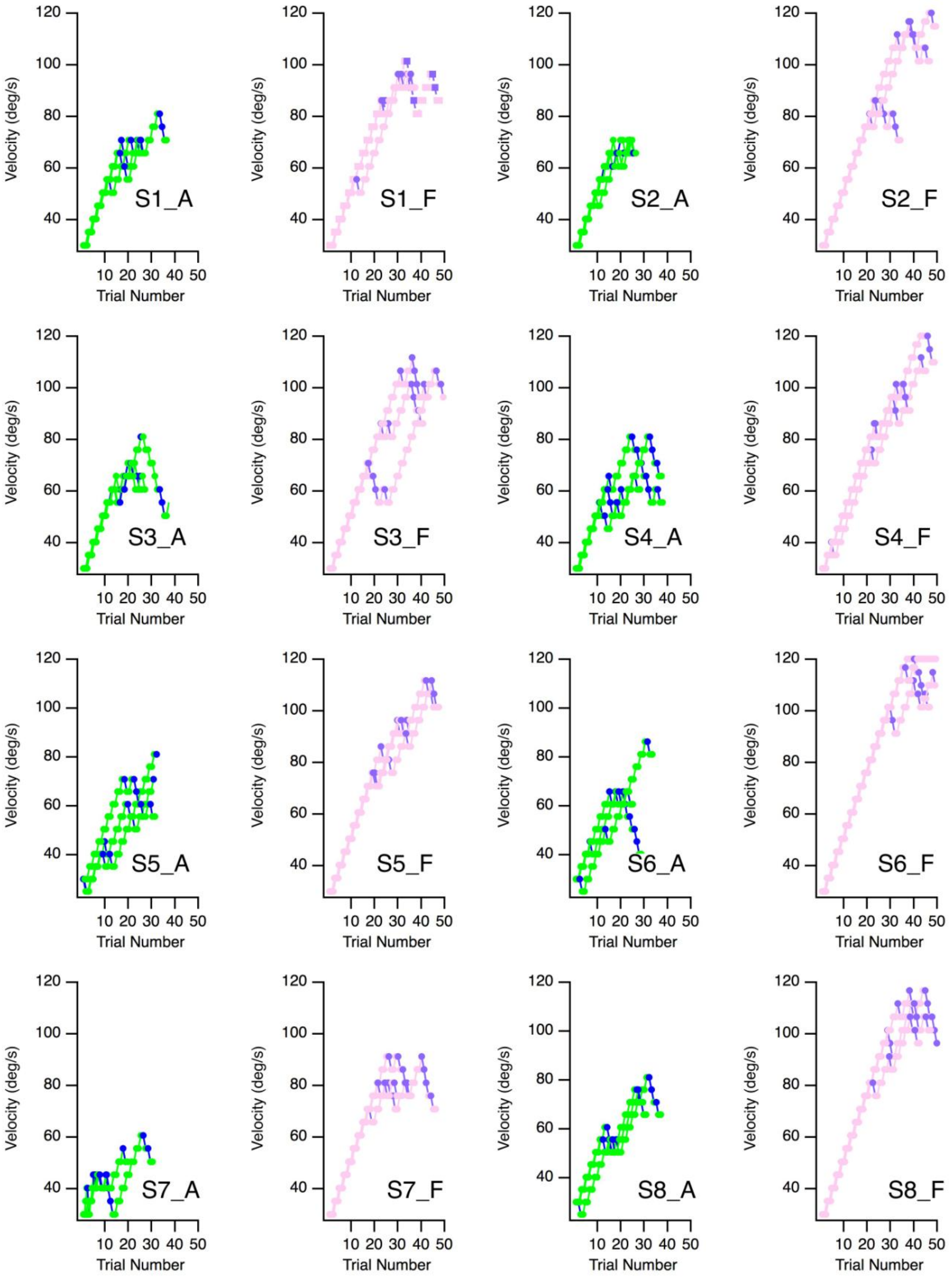

Supplementary Figure 1. Psychophysical Thresholds – Strabismic Participants. Staircase sequences for the motion salience task shown for each of the participants S1-S8 for the Amblyopic (_A) and Fellow (_F) eyes.
