# Supplementary material for: Impaired Activation of Visual Attention Network for Motion Salience Is Accompanied by Reduced Functional Connectivity between Frontal Eye Fields and Visual Cortex in Strabismic Amblyopia
Source: Front Hum Neurosci. 2017 Apr 21;11:195. doi: 10.3389/fnhum.2017.00195 (PMC5399630; doi:10.3389/fnhum.2017.00195)
Supplement: Supplementary file 8 [file Image2.PDF]

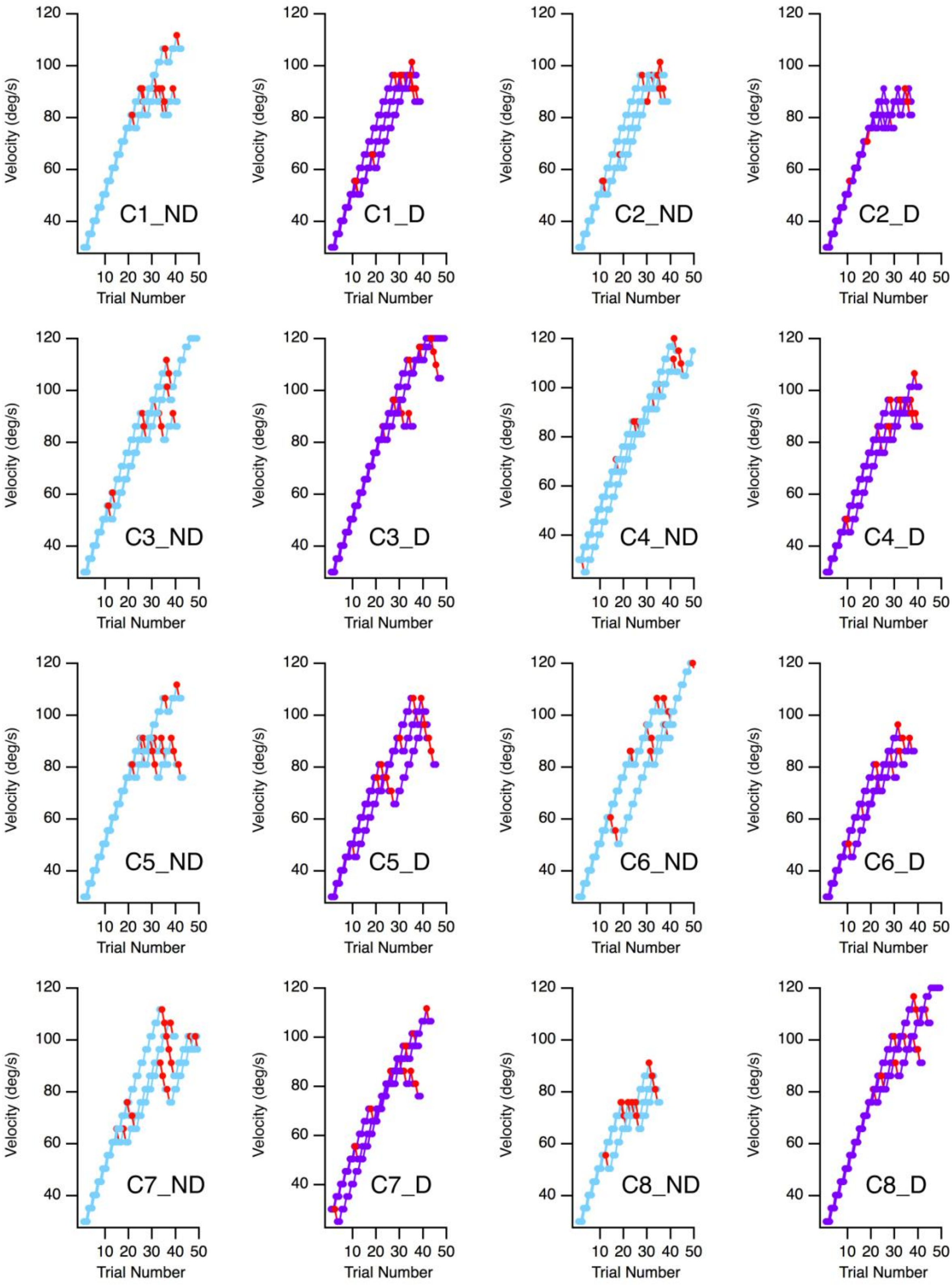

Supplementary Figure 2.Psychophysical Thresholds –Control Participants. Staircase sequences for the motion salience task shown for each of the participants C1-C8 for the Non-Dominant (_ND) and Dominant (_D) eyes.
